# Temporal Dynamics of Rhizosphere Communities Across the Life Cycle of *Panax notoginseng*

**DOI:** 10.3389/fmicb.2022.853077

**Published:** 2022-04-01

**Authors:** Guangfei Wei, Mengzhi Li, Guozhuang Zhang, Zhongjian Chen, Fugang Wei, Shuo Jiao, Jun Qian, Yong Wang, Jianhe Wei, Yitao Wang, Xiangxiao Meng, Martin Fitzgerald, Yuqi Yu, Linlin Dong, Shilin Chen

**Affiliations:** ^1^Key Laboratory of Beijing for Identification and Safety Evaluation of Chinese Medicine, Institute of Chinese Materia Medica, China Academy of Chinese Medical Sciences, Beijing, China; ^2^Institute of Sanqi Research, Wenshan University, Wenshan, China; ^3^Wenshan Miaoxiang Notoginseng Technology, Co., Ltd., Wenshan, China; ^4^State Key Laboratory of Crop Stress Biology in Arid Areas, College of Life Sciences, Northwest A&F University, Yangling, China; ^5^Hainan Provincial Key Laboratory of Resources Conservation and Development of Southern Medicine, Hainan Branch of the Institute of Medicinal Plant Development, Chinese Academy of Medical Sciences and Peking Union Medical College, Haikou, China; ^6^State Key Laboratory of Quality Research in Chinese Medicine, Institute of Chinese Medical Sciences, University of Macau, Taipa, Macao SAR, China; ^7^Ashdale Clinic, Cork, Ireland

**Keywords:** *Panax notoginseng*, rhizosphere microbiomes, developmental stages, temporal dynamics, life cycle

## Abstract

Rhizosphere microbiome promotes plant growth; however, the succession of rhizosphere microbial community during the growth stages of perennial medicinal plant *Panax notoginseng* (*P. notoginseng*) is still unclear. Here, amplicon sequencing was performed to assess the succession characteristics of rhizosphere microbiomes during developmental stages. Results showed that bacterial and fungal communities were mainly shaped by the development stages. The microbial α-diversities first increased and then decreased with plant growth and the variation in microbial composition was active at the 3-year root growth (3YR) stage. The variation trend of cross-domain co-occurrence network complexity was similar to that of α-diversities. Cross-domain nodes decreased at the 3YR stage and fungal nodes increased at the 3YR stage. This study provided a detailed and systematic survey of rhizosphere microbiomes during the growth stages of *P. notoginseng*. The findings revealed that the development stages of *P. notoginseng* drove the temporal dynamics of rhizosphere communities. This study helps in harnessing the power of microbiomes to evaluate herbal medicine growth and provides valuable information to guide the microbial breeding of medical plants.

## Introduction

Rhizosphere microbiome plays important role in nutrition acquisition, growth (Perez-Jaramillo et al., 2016), and pathogen resistance of plants (Liu et al., 2019). In return, plants affect the diversity, composition, structure, and function of the rhizosphere microbiome (Gomes et al., 2003; Lebreton et al., 2019). In *Arabidopsis thaliana* (*A. thaliana*), the diversity and composition of rhizosphere bacterial communities at the seedling stage significantly differ from those in other stages (vegetative, bolting, and flowering) (Chaparro et al., 2014). In soybeans, the vegetative growth stage significantly influences the structure of the bacterial communities and this effect persists at the late growth stage (Sugiyama et al., 2014). Variations in the composition of rhizodeposition allow plants to shape rhizosphere microbial communities for their benefit as a result of the selective power of plants (Tkacz et al., 2015). Therefore, understanding the succession of microbial communities in the rhizosphere during plant growth is of great significance for improving agricultural practices (Smalla et al., 2001).

Medicinal plants are essential for improving human health. Approximately, 75% of the population relies on herb-based medicines for routine healthcare in developing countries (Kim et al., 2015). Many medicinal plants are perennial, including transitions from seedling and flowering to aging annually. In *Panax ginseng*, the rhizosphere bacterial diversity decreases and fungal diversity increases at the root growth stage compared with those at vegetative, flowering, and fruiting stages (Dong et al., 2018b). The succession characteristics of rhizosphere microbiomes during the developmental stages of perennial medicinal plants may participate in mediating the accumulation of belowground biomass. However, to what extent a constant and beneficial rhizosphere community can be selected for a perennial medicinal plant during its developmental stages is still unknown.

*Panax notoginseng* (*P. notoginseng*) is a medicinal plant known as “Nanguo Shencao (miracle plant from South China)” and is used as the main raw material in Yunnan Baiyao and Xuesaitong due to its blood-invigorating effects (Kim et al., 2015). The therapeutic effects of *P. notoginseng* are mostly attributed to its bioactive saponin constituents, namely, notoginsenoside R1 and ginsenosides (Kim, 2012). As a valuable traditional medicine, this variety has a great annual demand in the global market (Li et al., 2020). Nevertheless, *P. notoginseng* suffers from replant problems with principal manifestations of low seed germination, poor seedling growth, and severe disease that led to yield reductions (Yang et al., 2015). The variations in rhizosphere microbiomes during plant growth might contribute to the replant problem of *P. notoginseng* (Luo et al., 2019). Continuous cropping could reduce the number of rhizobacteria and fungal diversity of *P. notoginseng* (Dong et al., 2016; Tan et al., 2017). Meanwhile, variations in the diversity and composition of the soil microbial community from a continuous cropping system could influence the soil productivity and yield of *P. notoginseng* (Li et al., 2020). *P. notoginseng* is a typical perennial plant mainly cultivated in the southwest of China (Meng et al., 2016). Owing to its perennial classification, this plant has annual vegetative, reproductive, and root growth stages. However, the succession of the rhizosphere microbial community during annual growth stages is still unclear.

In this study, high-throughput sequencing was performed to analyze the variations in microbial diversity, composition, and network structure to characterize the succession characteristics of rhizosphere microbiomes during the growth and development stages of *P. notoginseng*. We hypothesize that the temporal variations in rhizosphere communities are significantly driven by the development stages.

## Materials and Methods

### Sampling of Different Developmental Stages

**Figure 1:**
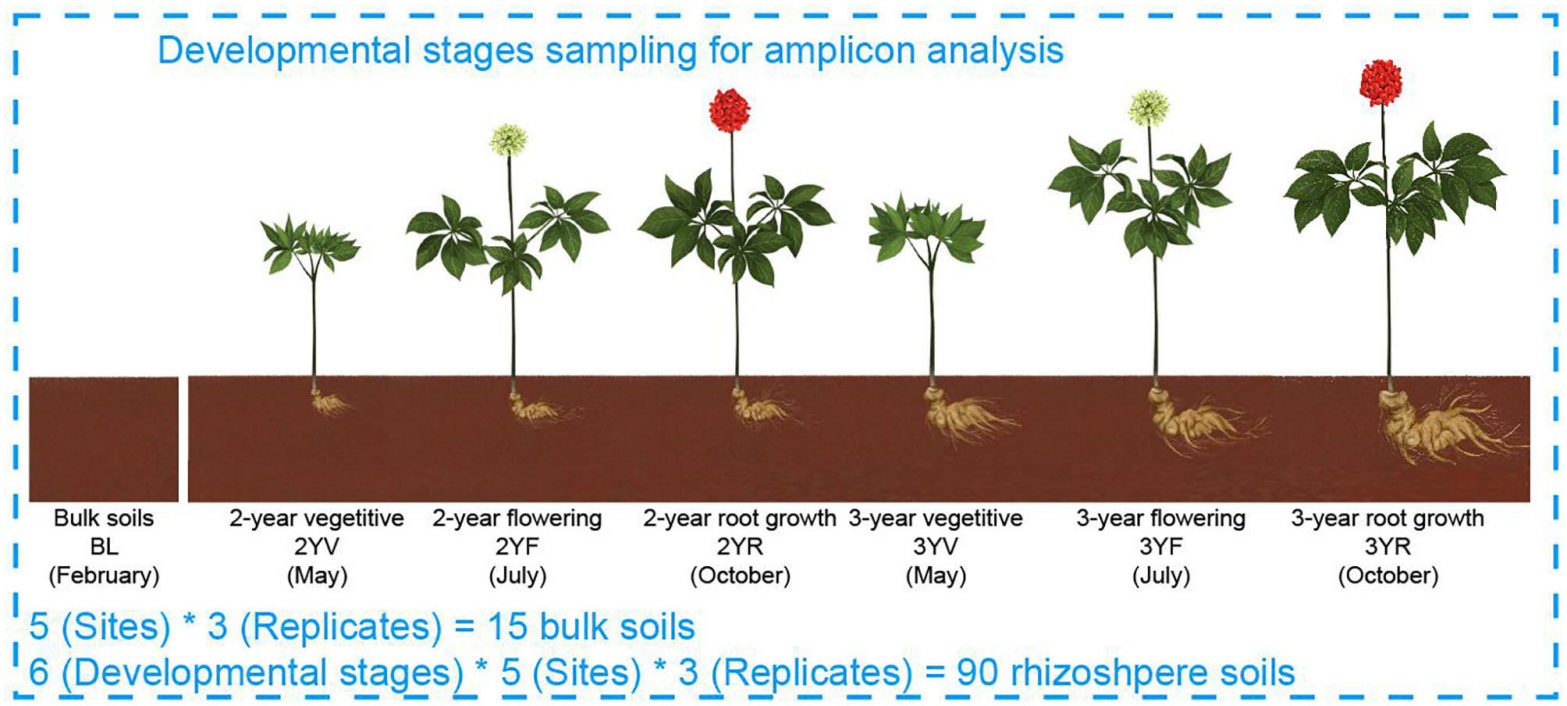
Model of experimental samples collection. BLs were collected from farmlands before *Panax notoginseng* (*P. notoginseng*) transplanting. Rhizosphere soil samples were collected from *P. notoginseng* plants.

Rhizosphere soil samples of *P. notoginseng* were collected at five fields in Yunnan Province of China during a 2-year growth from February 2016 to October 2017 (Figure 1; Supplementary Table S1). Briefly, 1-year-old seedlings were transplanted into each field and then cultivated in strict accordance with the Good Agricultural Practices (Heuberger et al., 2010; Zhang et al., 2021b). Every experimental field had three separated 1.4 m^2^ × 8.0 m^2^ plots as replicates. Before transplanting, 10 soil samples were collected from random locations in each plot and combined to generate one bulk soil (BL). The rhizosphere soil samples were then collected at the three developmental stages of *P. notoginseng* in each year: (1) vegetative (V, May); (2) flowering (F, Jul); and (3) root growth (R, Oct), namely, 2-year V (2YV), 2-year F (2YF), 2-year R (2YR), 3-year V (3YV), 3-year F (3YF), and 3-year R (3YR). At each sampling time, 10 randomly selected healthy plants were removed from each plot and mixed to generate one rhizosphere sample (Dong et al., 2018a). A total of 105 samples comprising 90 rhizosphere soil samples at six developmental stages and 15 unplanted BL samples in five fields were obtained, sieved (2 mm), and stored at −80°C for DNA extraction.

### Edaphic and Climatic Factors

Edaphic factors, namely, soil pH, organic matter (OM), available phosphate (AP), available potassium (AK), and total nitrogen (TN) were measured for the soil samples using standard test methods (Supplementary Table S1). The monthly mean temperature (MMT) of sampling time was acquired from the Worldclim database (www.worldclim.org/) using a geographic information system according to the longitude and latitude values of the collected samples (ArcGIS version 2.0) (Supplementary Table S1).

### Deoxyribonucleic Acid Extraction and Amplicon Sequencing of Soils at Different Developmental Stages

Total DNA from soil samples was extracted using the FastDNA SPIN Kit for Soil (MoBio Laboratories Incorporation, California, USA) to characterize the bacterial and fungal communities. 515F (5′-GTGCCAGCMGCCGCGG-3′)/907R (5′- CCGTCAATTCMTTTRAGTTT-3′) primer pair was used to amplify the V4–V5 region of bacterial 16S rRNA gene, and ITS1F (5′-CTTGGTCATTTAGAGGAAGTAA-3′)/ITS2R (5′- GCTGCGTTCTTCATCGATGC-3′) primer pair was used to amplify the fungal ITS region (Mueller et al., 2014; Jiao et al., 2019). Sequencing was performed on Illumina MiSeq PE 250 platform (Biozeron Corporation Ltd., Shanghai, China). The obtained paired-end sequences were demultiplexed, merged, and filtered using QIIME2 and USEARCH (Edgar, 2013; Bolyen et al., 2019). Chimeric sequences were then removed using the USEARCH (Edgar et al., 2011). The remaining sequences were split into operational taxonomic units (OTUs) with a 97% similarity level using the UPARSE pipeline (Edgar et al., 2011). Representative sequences of OTUs were assigned to taxonomic lineages using the Ribosomal Database Project (RDP) classifier against the SILVA database (release 132) for bacteria and UNITE database (release 7.1) for fungi (Quast et al., 2013; Nilsson et al., 2019). OTUs with less than two reads or fail to be aligned to the database (i.e., unclassified) were removed before further analysis. Finally, 16,033 bacterial operational taxonomic units (OTUs) and 5,147 fungal OTUs were obtained. The raw sequence data were uploaded to the National Center for Biotechnology Information (NCBI) Sequence Read Archive (SRA) under the bioproject number PRJNA559079. The relative abundance of OTUs was used in subsequence analyses except for the estimation of α-diversities and edgeR.

### Variations in Microbiome Diversity and Composition During Growth Stages

Operational taxonomic unit richness and Shannon index were calculated according to the rarefied sequence number to estimate the α-diversities of bacterial and fungal communities (bacteria: 44,332, fungi: 38,847) using the “rrarefy” and “diversity” functions in “vegan” package in *R* (Oksanen et al., 2019; Team RC, 2019). Linear least-square regression analysis with the second-order term was performed to fit the α-diversities to developmental stages using “lm” function. Nonparametric Kruskal–Wallis method was employed to further test the differences of α-diversities between different growth stages, followed by the Nemenyi test for multiple comparisons. Permutational multivariate ANOVA (PERMANOVA) was carried out to determine the effect size and significance of developmental stages on microbial compositions using “adonis” function in “vegan” package (Oksanen et al., 2019). Differentially abundant genera among microbial communities obtained from different stages were estimated by fitting a negative binomial generalized linear model in “edgeR” package (Robinson et al., 2010). After genera with CPM less than 100 in three samples were removed, “calcNormFactors” function was applied to normalize the library size according to the trimmed mean of the M value method. Common and tagwise dispersions were obtained using the “estimateDisp” function with a design matrix. The “glmFit” function was employed to fit a generalized linear model with a negative binomial distribution, and differential abundant genera were tested using the likelihood ratio test (glmLRT function). *P*-values were further corrected using the “BH” method (Benjamini and Hochberg, 1995).

### Co-occurrence Network Construction at Different Developmental Stages

A cross-domain co-occurrence network was first constructed for each developmental stage based on correlations to reveal the variations in potential interaction patterns of rhizosphere microbiomes during *P. notoginseng* growth. Only bacterial and fungal OTUs with a relative abundance >0.01%, and spearman correlations > 0.7 or < −0.7 with the false discovery rate (FDR) corrected *P* < 0.01 were used to construct the network (Zhang et al., 2021b). A set of node-level and network-level topological properties were then calculated in the “igraph” package in *R* (Ma et al., 2017). The node-level feature set included degree centrality (the number of adjacent edges), betweenness centrality (the number of shortest paths going through a node), closeness centrality (the number of steps required to access all other nodes from a given node), and eigenvector centrality (the tendency for a certain node to share connections with other nodes that link to many other taxa). These node topologies potentially represent the role of nodes in maintaining co-occurrence patterns (Jiao et al., 2017; Zhang et al., 2021b). The network-level feature set consisted of complexity measures (i.e., graph density, average path length, and clustering coefficient) and stability measures (i.e., natural connectivity) (Jun et al., 2010; Ma et al., 2016; Fan et al., 2018; Zhang et al., 2021a). Wilcox rank-sum test was performed to compare the node-level properties between bacterial and fungal nodes. Bacterial and fungal subnetworks at each developmental stage were further extracted using the “igraph” package in *R*.

### Construction of Redundancy Analysis and Structural Equation Model at Different Developmental Stages

Redundancy analysis was performed to assess the influence of developmental stages and environmental factors on bacterial and fungal communities using the “rda” function in the “vegan” package (Oksanen et al., 2019). Constrained analysis of principal coordinates (CAP) was then conducted to visualize the succession of bacterial and fungal communities based on Bray–Curtis dissimilarities using “capscale” and “cmdscale” functions in the “vegan” package (Oksanen et al., 2019).

A structural equation model was also established to further explore the potential causal relationships among biotic and abiotic factors using the “lavaan” package in *R* (Rosseel, 2012). The representative of variations in microbial communities were the axes of principal component analysis based on scaled variables represented edaphic factors and the axes of principal coordinate analysis (PCoA) based on Bray–Curtis dissimilarities. The goodness of fit of the two models was assessed using chi-square statistics and root mean square error of approximation (RMSEA).

## Results

### Temporal Dynamics of Microbial Diversities During *P. notoginseng* Developmental Stages

**Figure 2:**
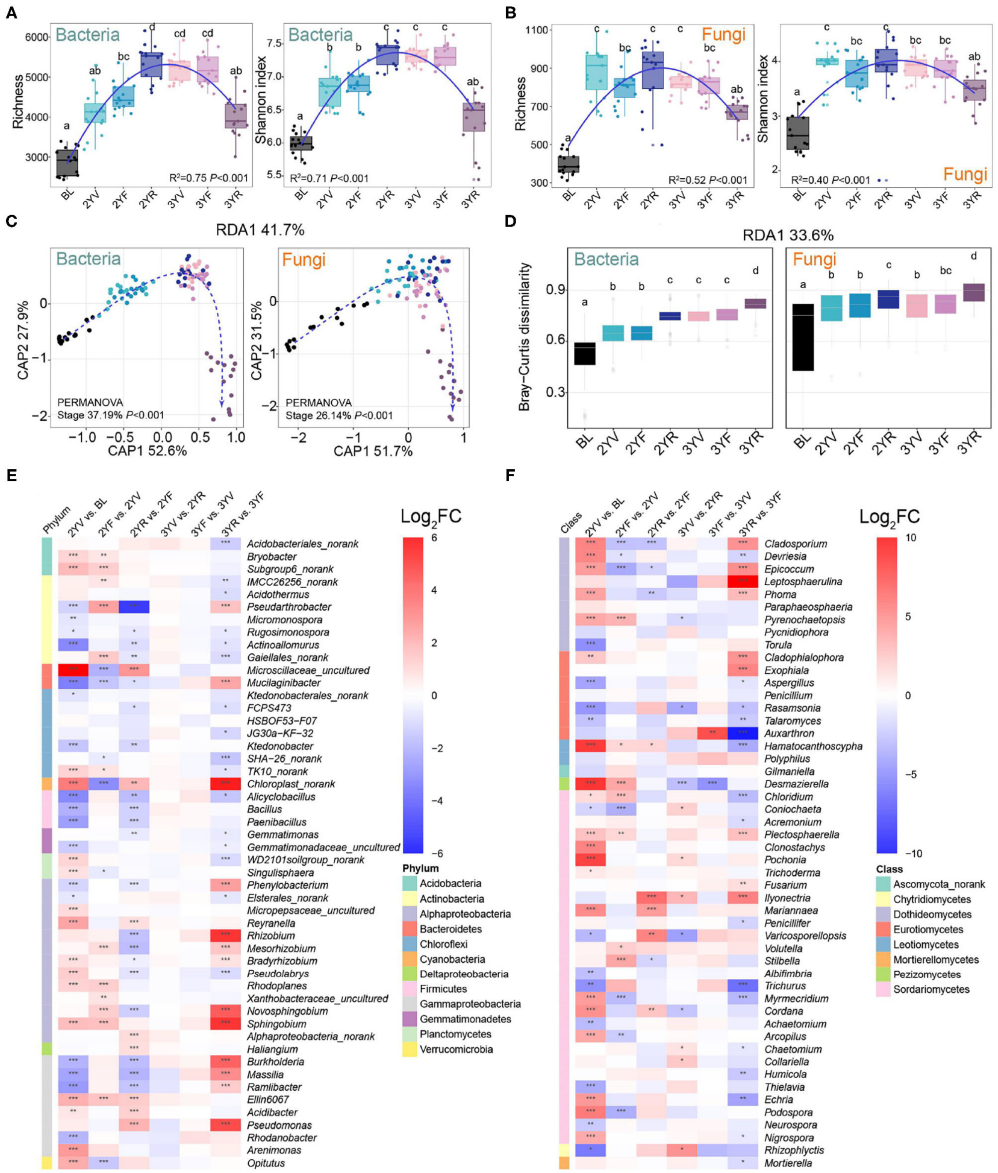
Changes in α- and β-diversities of bacterial and fungal communities across developmental stages of *P. notoginseng*. Bacterial **(A)** and fungal **(B)** α-diversities at different growth stages are represented by operational taxonomic unit (OTU) richness and Shannon index. BL, 2YV, 2YF, 2YR, 3YV, 3YF, and 3YR represent BLs and the 2-year vegetative, 2-year flowering, 2-year root growth, 3-year vegetative, 3-year flowering, and 3-year root growth stages, respectively. **(C)** Variation of bacterial and fungal communities constrained to developmental stages based on principal coordinates analysis (CAP). **(D)** Bray–Curtis distances between BLs and rhizosphere soils of other stages. The enrichment and depletion of bacterial **(E)** and fungal **(F)** genera with the average relative abundance of the top 50. Color represents log-transformed fold change. Different letters denote significance between compared groups (*P* < 0.05). *P < 0.05; **P < 0.01; ***P < 0.001.

The succession characteristics of bacterial and fungal α-diversities during the developmental stages were analyzed using OTU richness and Shannon indices (Figures 2A,B). Linear least-square regression with second-order term showed that the α-diversities of bacteria and fungi were parabolic with plant growth (richness: *R*^2^ = 0.75, *P* < 0.001 for bacteria; *R*^2^ = 0.52, *P* < 0.001 for fungi. Shannon indices: *R*^2^ = 0.71, *P* < 0.001 for bacteria; *R*^2^ = 0.40, *P* < 0.001 for fungi). Compared with those of BLs, the values of *P. notoginseng* for rhizosphere bacterial and fungal communities increased significantly. During *P. notoginseng* growth, the bacterial α-diversities increased gradually in soil from the 2YV stage to the 2YR stage. Meanwhile, the fungal α-diversities peaked at the 2YV stage and then fluctuated mildly until the 3YF stage. Interestingly, compared to the 3YF stage, the richness and Shannon indices of soil bacterial and fungal communities at the 3-year-root growth (3YR) stage were remarkably reduced. These results revealed the α-diversities of rhizosphere communities first increased and then decreased with plant growth and the dynamic succession characteristics were similar for bacterial and fungal communities.

Permutational multivariate ANOVA (PERMANOVA) based on Bray–Curtis dissimilarities revealed that the developmental stages significantly drove the variations in the bacterial (*R*^2^ = 37.19%, *P* < 0.001) and fungal (*R*^2^ = 26.14%, *P* < 0.001) β-diversities (Figure 2C). Constrained analysis of principal coordinates showed a distinct separation among different stages, and the samples of the 3YR stage could be separated from the others. Bray–Curtis dissimilarities between rhizosphere soils and BLs increased with incremental growth (Figure 2D). These results indicated that the biodiversity and community structure of rhizosphere bacterial and fungal communities at different stages of host plant development might be significantly related to the succession of host plant characteristics.

### Temporal Dynamics of Microbial Compositions During *P. notoginseng* Developmental Stages

The microbial compositions in the rhizosphere soil of *P. notoginseng* also changed with the developmental stages (Figures 2E,F; Supplementary Figures S1,S2). Compared with those in BLs, the relative abundance of bacterial genera, namely, *Bacillus, Paenibacillus*, and other genera from Firmicutes largely decreased (Figure 2E). At the 3YR harvest stage, the relative abundance of Cyanobacteria and certain bacterial genera, namely, *Dyadobacter, Dysgonomonas, Burkholderia, Delftia, Flavobacterium, Lechevalieria*, and *Hyphomicrobium* increased significantly. Some fungal genera such as *Cladosporium, Epicoccum, Leptosphaerulina, Phoma, Cladophialophora, Exophiala, Fusarium, Plectosphaerella*, and *Ilyonectria* were enriched at the 3YR stage (Figure 2F). These results revealed that the composition variations of bacterial and fungal communities were active at the 3YR stage.

### Variations in Co-occurrence Patterns During *P. notoginseng* Developmental Stages

**Figure 3:**
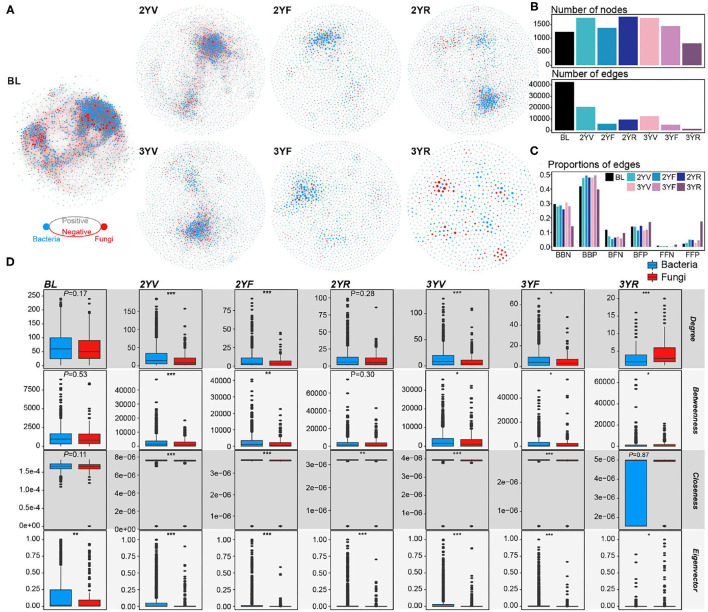
Cross-domain networks and node-level topological properties. **(A)** Cross-domain networks are constructed from each developmental stage. Blue and red nodes represent bacterial and fungal nodes, respectively. Gray and red edges represent positive and negative correlations among nodes, respectively. **(B)** The number of nodes and edges in each cross-domain network. **(C)** The proportion of the different types of edges in each cross-domain network. **(D)** Node-level topological properties of bacterial and fungal nodes in each cross-domain network. *, *P* < 0.05; **, *P* < 0.01; ***, *P* < 0.001.

**Figure 4:**
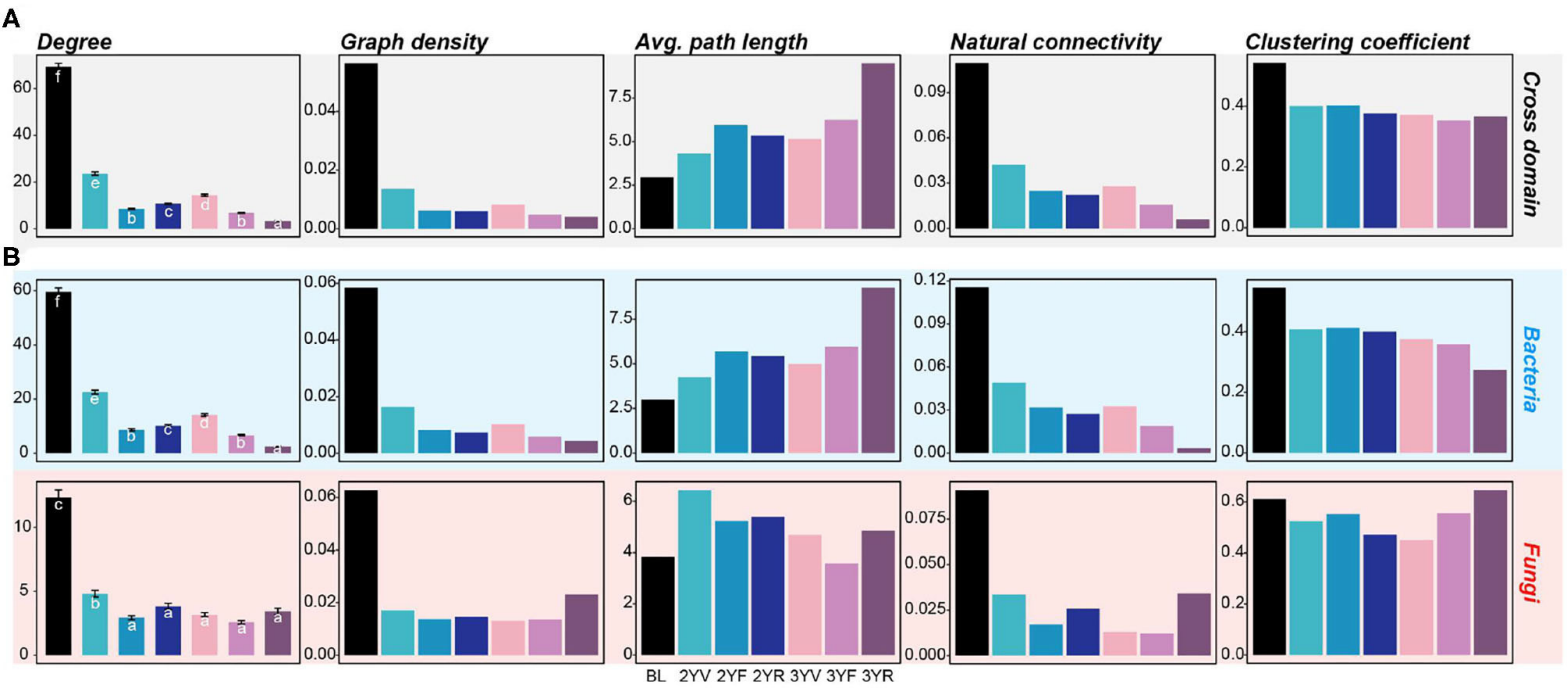
Network-level topological properties of cross-domain and single-domain networks are constructed from each developmental stage. **(A)** Network-level topologies of cross-domain networks. **(B)** Network-level topologies of bacterial and fungal subnetworks. The colors of the bars represent different developmental stages. Different letters denote significance between the compared groups (*P* < 0.05).

According to the cross-domain co-occurrence network constructed from each developmental stage, the variation trend of node number was similar to that of α-diversities (Figures 3A, 4A). However, the number of edges of rhizosphere networks was lower than that of the network obtained from BL, and the 3YR network exhibited the lowest number of edges (Figure 3B). The network-level topological properties, namely, average degree, graph density, natural connectivity, and clustering coefficient, also decreased after plant cultivation (Figure 4A). The average path lengths of rhizosphere networks were higher than those of BL networks (Figure 4A). In addition, the topologies of the 3YR network showed dramatic changes compared with those in previous stages (Figures 3A,B).

For the proportions of different edge types, the proportion of negative edges decreased in the rhizosphere soil compared with that in the BL (Figure 3C). At the 3YR stage, the ratios of bacteria–bacteria negative (BBN) edges and bacteria–bacteria positive (BBP) edges declined and those of fungi-associated links, especially fungi–fungi positive (FFP) edges increased. In the BL network, the node-level properties of bacterial and fungal nodes only exhibited slight differences (Wilcoxon rank-sum test, *P* > 0.05), except for the eigenvector centrality (Wilcoxon rank-sum test, *P* < 0.05; Figure 3D). After plant cultivation, the topological centralities of bacterial nodes became significantly higher than those of fungal nodes until the 3YF stage. The fungal nodes showed remarkably higher centrality values than the bacterial nodes at the 3YR stage (Wilcoxon rank-sum test, *P* < 0.05), except for the closeness centrality (Wilcoxon rank-sum test, *P* = 0.87).

For the single-domain subnetworks, the topological characters of bacterial networks showed consistent trends with the cross-domain networks (Figure 4B; Supplementary Figure S3). However, the fungal network exhibited an opposite trend at the 3YR stage, that is, the graph density, natural connectivity, and clustering coefficient of the fungal network at the 3YR stage increased compared with those of the networks of previous developmental stages. These results showed that the network variations of bacterial and fungal communities were active at the 3YR stage.

### Microbial Communities Driven by the Development Stages of *P. notoginseng*

**Figure 5:**
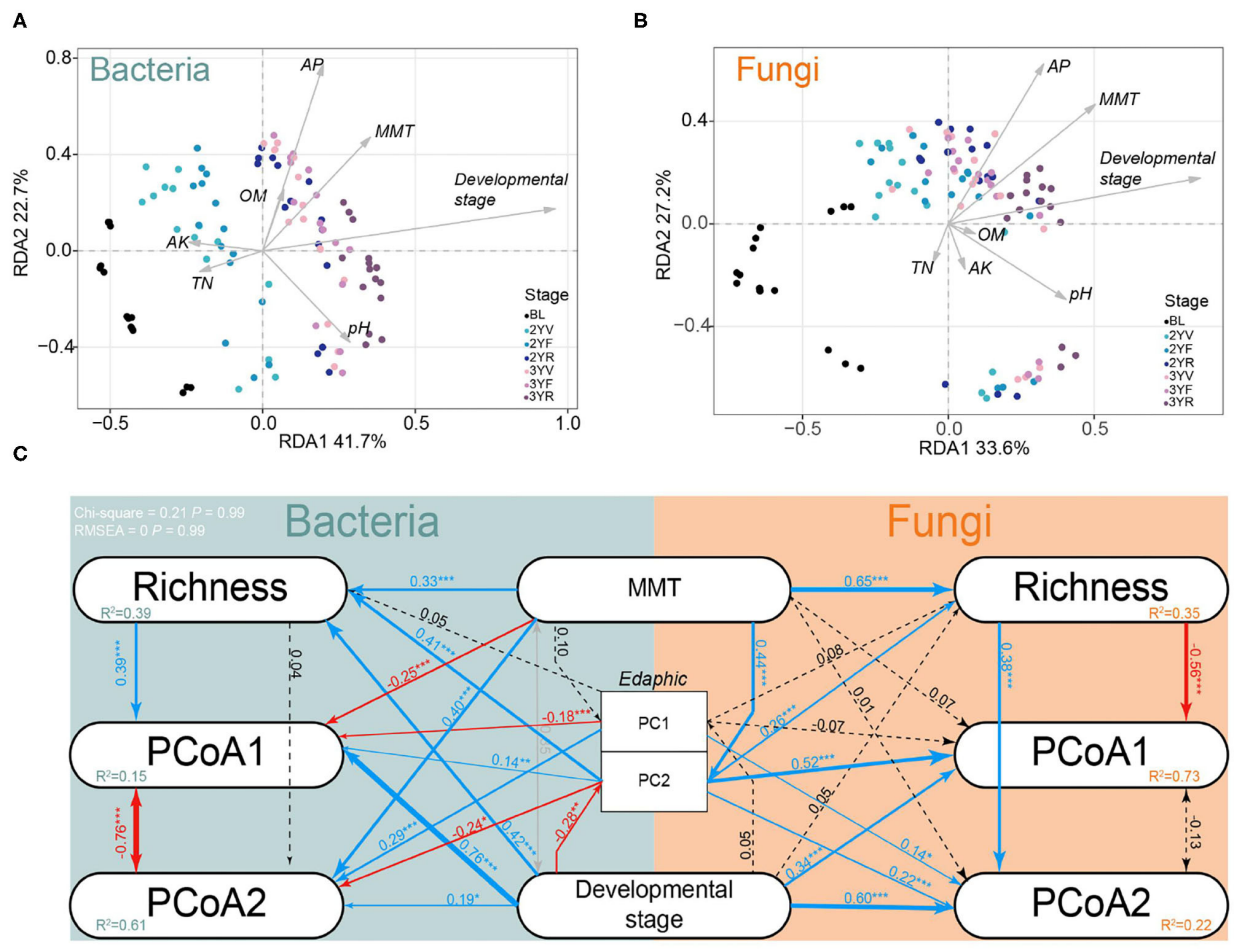
Redundancy analysis (RDA) and structural equation model (SEM) in developmental stages sampling dataset. Effects of climatic factors, edaphic factors, and developmental stages on bacterial **(A)** and fungal **(B)** communities using RDA. MMT, month mean temperature; AK, available potassium; OM, organic matter; AP, available phosphate; TN, total nitrogen. **(C)** Effects of climatic factors, edaphic factors, and developmental stages on rhizosphere communities using SEM. PC1 and PC2 represent the first and second axes of principal coordinate analysis (PCoA) of edaphic factors; PCoA1 and PCoA2 represent the first and second axes of PCoA based on Bray–Curtis distances; Richness: OTU richness. MMT: month mean temperature. Solid lines represent significant (*P* < 0.1) path coefficients (blue, positive; red, negative), and dotted lines represent paths with *P* ≥ 0.1. The gray line represents the correlation between independent variables. *R*^2^ represents variance explained by the model.

Monthly mean temperature (MMT), edaphic factors, and developmental stages were identified as significant drivers that influenced the rhizosphere microbiomes. RDA was used to preliminarily characterize the effects of environmental factors and developmental stages on rhizosphere microbiomes during plant growth (Figures 5A,B; Supplementary Table S2). The results showed that the developmental stages were the most important variables in bacterial (6.76%, *P* < 0.001) and fungal communities (5.26%, *P* < 0.001).

A SEM was then constructed to evaluate the most influential factors of the variance in microbial communities during plant growth (Figure 5C). The developmental stages were found to have a significant direct effect on PCoA1 (bacteria, 0.76, *P* < 0.001; fungi, 0.34, *P* < 0.001), PCoA2 (bacteria, 0.19, *P* < 0.05; fungi, 0.60, *P* < 0.001), and bacterial richness (0.42, *P* < 0.001). An indirect positive effect of development stages on PCoA1 (*R* = 0.39, *P* < 0.001) was also found *via* their impacts on richness (*R* = 0.420, *P* < 0.001). These data further emphasized that the developmental stages of *P. notoginseng* could significantly drive the rhizosphere microbiomes.

## Discussion

Here, we used amplicon sequencing to determine the temporal dynamics of diversity, composition, and network structures of bacterial and fungal communities during the overall growth stages of *P. notoginseng*. Microbial biodiversity is important to ecosystem functions involved in plant growth and health (Garland et al., 2021; Jiao et al., 2022). In this study, the bacterial and fungal α-diversities exhibited increasing trends in the rhizosphere soil of *P. notoginseng* compared with those in the BL. The higher α-diversity in rhizosphere soil than that in BL was also observed in cotton (Qiao et al., 2017). Compared with the bare BL, a large amount of rhizodeposits, namely, root exudates, cells, and mucilage, existed in the rhizosphere due to root activity (Philippot et al., 2013). These diverse and abundant sources might provide additional ecological niches, thus improving the species' coexistence and biodiversity in the rhizosphere (Kraft et al., 2014). Meanwhile, the bacterial and fungal α-diversities increased and then decreased during *P. notoginseng* growth, a trend similarly observed in *P. ginseng* (Xiao et al., 2016) and *Pseudostellaria heterophylla* (Zhao et al., 2016). Constrained analysis of principal coordinates showed a clear boundary between bacterial and fungal β-diversities in the BL and rhizosphere soil samples. This finding indicated that rhizosphere bacterial and fungal community structures might have a host-selective effect (Berg and Smalla, 2009; Uroz et al., 2010). Rhizosphere bacterial β-diversities also varied during the development stages of *P. notoginseng* and a similar trend was found in *A. thaliana* (Chaparro et al., 2014). Rhizodeposits, which are the result of plant activity, act as resources and selective agents for the rhizosphere microbial communities. For example, the 1-deoxy-L-erythritol and glycerol-gulo-hepto released by *A. thaliana* might be correlated with the specific rhizosphere recruitment of *Pseudomonadales* (Carvalhais et al., 2015). In addition, the effects of the plant on rhizosphere microbial communities might also reflect the host functional requirements for soil microbes shaped in the long history of plant–microbe coevolution (Foster et al., 2017). A typical example is that the soybean plant recruits microbes to the rhizosphere depending on their functions associated with plant nutrient acquisition (Mendes et al., 2014). Thus, the covariation of bacterial and fungal communities during plant growth might be due to the changes in plant trait expression and functional requirements across different growth stages (Foster et al., 2017; Zhalnina et al., 2018). The variation in microbial composition (bacterial genera, *Dyadobacter* and *Dysgonomonas*; fungal genera, *Cladosporium, Fusarium, Phoma*, and *Cladophialophora*) was active at the 3YR stage. This finding is within expectation because the roots of *P. notoginseng* swell significantly at this stage, indicating the strong root activity and requirements for nutrients which can impose strong effects on the rhizosphere microbiomes (Berg and Smalla, 2009; Uroz et al., 2010). For instance, a sharp increase in Cyanobacteria at the 3YR stage was observed. Cyanobacteria has a strong ability to colonize plant roots and promote plant growth to meet the needs of rapid root growth at this stage (Franche et al., 2008). In summary, these results indicated that *P. notoginseng* could potentially select a set of microbes and build-up succession characteristics according to its growth needs, and the strength of its rhizosphere effect varies with its growth.

Microbial interactions in the rhizosphere also have great influences on rhizosphere functions and plant health (Toju et al., 2018; Jiao et al., 2022). Network analysis showed that the rhizosphere soil exhibited lower network complexity and ratios of negative links than the unplanted BL; a similar finding was observed in soybean and wheat (Mendes et al., 2014; Fan et al., 2018). Approximately, 20% of the carbon fixed by plant photosynthesis was transferred to the rhizosphere soil through root activity (Huang et al., 2019). These rhizodeposits might decrease the network complexity and negative inter-microbe links in two ways. On one hand, these additional resources reduce competition (Costello et al., 2012; Fan et al., 2018). On the other hand, in contrast to the complex substrates in BL, the root exudates are rich in simple compounds, such as sugars, amino acids, and aliphatic acids, which could weaken the metabolic links among microbes (Bai et al., 2015; Xu et al., 2018). In addition, network stability dramatically declined after plant cultivation; this phenomenon was also observed in the intestinal microbiota after antibiotic treatment (Ruiz et al., 2017). The loss of network stability indicated that the root exudates might also act as potential disturbance factors in shaping rhizosphere microbiomes. At the 3YR stage, the complexity and stability of the bacterial network experienced a second sharp decline, whereas the corresponding properties of the fungal network at this stage showed an increase. This phenomenon might be due to the higher resistance of fungal interactions to potential environmental disturbance compared with bacteria, which was consistent with the less stable bacterial network than the fungal network under drought stress (de Vries et al., 2018). The ratio of inter-fungi positive links increased sharply at this stage, indicating that the root activities improve the among-fungi mutualistic interactions or covariations in response to the environment (Shi et al., 2016). By contrast, the collapse of the bacterial network at this stage indicated that the plant has weakened the among-bacteria links in terms of signal or resource (Faust and Raes, 2012). Decreased bacterial activities could also contribute to weak interactions (Shi et al., 2016). The cross-domain interactions, especially the positive links between bacteria and fungi, showed an increase at the last growth stage that might have resulted from the bacterial taxa that tend to interact with fungi, such as “fungi-feeders” (Frey-Klett et al., 2011; Ballhausen and de Boer, 2016). In summary, the network-based result emphasized the differences between the potential bacterial and fungal interactions, especially in terms of their responses to plants at the 3YR stage. This finding contrasted sharply with the similar diversity patterns of the two domains and provided a new dimension regarding the succession of rhizosphere microbiomes during *P. notoginseng* growth. The complexity of species interaction network in the rhizosphere (especially bacteria) might have a positive correlation with resistance to pathogen invasion (Case, 1990; Wei et al., 2015). A previous study on common beans also showed that the pathogen-resistant cultivar exhibits a more complex network in the rhizosphere compared with pathogen-susceptible cultivars (Mendes et al., 2018). Thus, the collapse of bacterial network structure after plant cultivation and at the 3YR stage might lead to the replant problem of *P. notoginseng*.

## Conclusion

In conclusion, the plant developmental stage was a main significant driving force affecting the rhizosphere microbiomes. The succession characteristics of bacterial and fungal diversities showed similar parabolic patterns during plant growth, thus reflecting the adaptability of plant microbial communities to changes during plant development. The complexity and stability in co-occurrence patterns of rhizosphere microbiomes decreased during plant growth. This work provides a comprehensive understanding of predicting the response of microbial communities in plant growth.

## Data Availability Statement

The datasets presented in this study can be found in online repositories. The names of the repository/repositories and accession number(s) can be found in the article/Supplementary Material.

## Author Contributions

GW and LD analyzed the data and drafted the manuscript. GW, ML, and GZ performed the wet-lab experiments. ZC, FW, and SJ collected the samples. JQ and YW analyzed the data and revised the manuscript critically. JW, YW, XM, MF, YY, LD, and SC coordinated the study, granted funds, and participated in the drafting and revision of the manuscript. All authors read and approved the final manuscript.

## Conflict of Interest

FW and YY were employed by Wenshan Miaoxiang Notoginseng Technology Co., Ltd. The remaining authors declare that the research was conducted in the absence of any commercial or financial relationships that could be construed as a potential conflict of interest.

## Publisher's Note

All claims expressed in this article are solely those of the authors and do not necessarily represent those of their affiliated organizations, or those of the publisher, the editors and the reviewers. Any product that may be evaluated in this article, or claim that may be made by its manufacturer, is not guaranteed or endorsed by the publisher.

## References

[B1] Bai Y. Muller D. B. Srinivas G. Garrido-Oter R. Potthoff E. Rott M. (2015). Functional overlap of the Arabidopsis leaf and root microbiota. Nature. 528, 364–369. 10.1038/nature16192 26633631

[B2] Ballhausen M.-B. de Boer W. (2016). The sapro-rhizosphere: carbon flow from saprotrophic fungi into fungus-feeding bacteria. Soil Biol. Biochem. 102, 14–17. 10.1016/j.soilbio.2016.06.014

[B3] Benjamini Y. Hochberg Y. (1995). Controlling the false discovery rate: a practical and powerful approach to multiple testing. J. R. Stat. Soc. Ser. B. 57, 289–300. 10.1111/j.2517-6161.1995.tb02031.x

[B4] Berg G. Smalla K. (2009). Plant species and soil type cooperatively shape the structure and function of microbial communities in the rhizosphere. FEMS Microbiol Ecol. 68, 1–13. 10.1111/j.1574-6941.2009.00654.x 19243436

[B5] Bolyen E. Rideout J. R. Dillon M. R. Bokulich N. A. Abnet C. C. Al-Ghalith G. A. (2019). Reproducible, interactive, scalable and extensible microbiome data science using QIIME 2. Nat. Biotechnol. 37, 852–857. 10.1038/s41587-019-0209-9 31341288 PMC7015180

[B6] Carvalhais L. C. Dennis P. G. Badri D. V. Kidd B. N. Vivanco J. M. Schenk P. M. (2015). Linking jasmonic acid signaling, root exudates, and rhizosphere microbiomes. Mol Plant Microbe Interact. 28, 1049–1058. 10.1094/MPMI-01-15-0016-R 26035128

[B7] Case T. J. (1990). Invasion resistance arises in strongly interacting species-rich model competition communities. Proc Natl Acad Sci USA. 87, 9610–9614. 10.1073/pnas.87.24.9610 11607132 PMC55222

[B8] Chaparro J. M. Badri D. V. Vivanco J. M. (2014). Rhizosphere microbiome assemblage is affected by plant development. ISME J. 8, 790–803. 10.1038/ismej.2013.196 24196324 PMC3960538

[B9] Costello E. K. Stagaman K. Dethlefsen L. Bohannan B. J. Relman D. A. (2012). The application of ecological theory toward an understanding of the human microbiome. Science. 336, 1255–1262. 10.1126/science.1224203 22674335 PMC4208626

[B10] de Vries Griffiths F. T. Bailey R. I. Craig M. Girlanda H. Gweon M. (2018). Soil bacterial networks are less stable under drought than fungal networks. Nat Commun. 9, 3033. 10.1038/s41467-018-05516-7 30072764 PMC6072794

[B11] Dong L. Xu J. Feng G. Chen X. Li S. (2016). Soil bacterial and fungal community dynamics in relation to *Panax notoginseng* death rate in a continuous cropping system. Sci Rep. 6, 31802. 10.1038/srep31802 27549984 PMC4994099

[B12] Dong L. Xu J. Li Y. Fang H. Niu W. Li X.. (2018a). Manipulation of microbial community in the rhizosphere alleviates the replanting issues in Panax ginseng. Soil Biol. Biochem. 125, 64–74. 10.1016/j.soilbio.2018.06.028

[B13] Dong L. Xu J. Zhang L. Cheng R. Wei G. Su H. (2018b). Rhizospheric microbial communities are driven by *Panax ginseng* at different growth stages and biocontrol bacteria alleviates replanting mortality. Acta Pharm. Sin. B. 8, 272–282. 10.1016/j.apsb.2017.12.011 29719788 PMC5925392

[B14] Edgar R. C. (2013). UPARSE highly accurate OTU sequences from microbial amplicon reads. Nat. Methods. 10, 996–998. 10.1038/nmeth.2604 23955772

[B15] Edgar R. C. Haas B. J. Clemente J. C. Quince C. Knight R. (2011). UCHIME improves sensitivity and speed of chimera detection. Bioinformatics. 27, 2194–2200. 10.1093/bioinformatics/btr381 21700674 PMC3150044

[B16] Fan K. Weisenhorn P. Gilbert J. A. Chu H. (2018). Wheat rhizosphere harbors a less complex and more stable microbial co-occurrence pattern than bulk soil. Soil Biol. Biochem. 125, 251–260. 10.1016/j.soilbio.2018.07.022

[B17] Faust K. Raes J. (2012). Microbial interactions: from networks to models. Nat. Rev. Microbiol. 10, 538–550. 10.1038/nrmicro2832 22796884

[B18] Foster K. R. Schluter J. Coyte K. Z. Rakoff-Nahoum S. (2017). The evolution of the host microbiome as an ecosystem on a leash. Nature. 548, 43–51. 10.1038/nature23292 28770836 PMC5749636

[B19] Franche C. Lindström K. Elmerich C. (2008). Nitrogen-fixing bacteria associated with leguminous and non-leguminous plants. Plant Soil. 321, 35–59. 10.1007/s11104-008-9833-8

[B20] Frey-Klett P. Burlinson P. Deveau A. Barret M. Tarkka M. Sarniguet A. (2011). Bacterial-fungal interactions: hyphens between agricultural, clinical, environmental, and food microbiologists. Microbiol. Mol. Biol. Rev. 75, 583–609. 10.1128/MMBR.00020-11 22126995 PMC3232736

[B21] Garland G. Edlinger A. Banerjee S. Degrune F. García-Palacios P. Pescador D. S. (2021). Crop cover is more important than rotational diversity for soil multifunctionality and cereal yields in European cropping systems. Nat. Food. 2, 28–37. 10.1038/s43016-020-00210-8 37117662

[B22] Gomes N. C. Fagbola O. Costa R. Rumjanek N. G. Buchner A. Mendona-Hagler L. (2003). Dynamics of fungal communities in bulk and maize rhizosphere soil in the tropics. Appl. Environ. Microbiol. 69, 3758–3766. 10.1128/AEM.69.7.3758-3766.2003 12839741 PMC165189

[B23] Heuberger H. Bauer R. Friedl F. Heubl G. Hummelsberger J. Nogel R. (2010). Cultivation and breeding of Chinese medicinal plants in Germany. Planta Med. 76, 1956–1962. 10.1055/s-0030-1250528 21077027

[B24] Huang A. C. Jiang T. Liu Y. X. Bai Y. C. Reed J. Qu B. A. (2019). A specialized metabolic network selectively modulates *Arabidopsis* root microbiota. Science 364, eaau6389. 10.1126/science.aau6389 31073042

[B25] Jiao S. Chen W. Wei G. (2017). Biogeography and ecological diversity patterns of rare and abundant bacteria in oil-contaminated soils. Mol. Ecol. 26, 5305–5317. 10.1111/mec.14218 28665016

[B26] Jiao S. Lu Y. Wei G. (2022). Soil multitrophic network complexity enhances the link between biodiversity and multifunctionality in agricultural systems. Glob Chang. Biol. 28, 140–153. 10.1111/gcb.15917 34610173

[B27] Jiao S. Xu Y. Zhang J. Hao X. Lu Y. (2019). Core microbiota in agricultural soils and their potential associations with nutrient cycling. mSystems. 4, e00313–e00318. 10.1128/mSystems.00313-18 30944882 PMC6435817

[B28] Jun W. Barahona M. Yue-Jin T. Hong-Zhong D. (2010). Natural connectivity of complex networks. Chin. Phys. Lett. 2010, 27. 10.1088/0256-307X/27/7/078902

[B29] Kim D. H. (2012). Chemical diversity of *Panax ginseng, Panax quinquifolium*, and *Panax notoginseng*. J Ginseng Res. 36, 1–15. 10.5142/jgr.2012.36.1.1 23717099 PMC3659563

[B30] Kim Y. J. Zhang D. Yang D. C. (2015). Biosynthesis and biotechnological production of ginsenosides. Biotechnol Adv. 33, 717–735. 10.1016/j.biotechadv.2015.03.001 25747290

[B31] Kraft N. J. B. Adler P. B. Godoy O. James E. C. Fuller S. Levine J. M. (2014). Community assembly, coexistence and the environmental filtering metaphor. Funct. Ecol. 29, 592–599. 10.1111/1365-2435.1234530135118

[B32] Lebreton L. Guillerm-Erckelboudt A. Y. Gazengel K. Linglin J. Ourry M. Glory P. (2019). Temporal dynamics of bacterial and fungal communities during the infection of Brassica rapa roots by the protist *Plasmodiophora brassicae*. PLoS ONE. 14, e0204195. 10.1371/journal.pone.0204195 30802246 PMC6388920

[B33] Li M. Chen Z. Qian J. Wei F. Zhang G. Wang Y.. (2020). Composition and function of rhizosphere microbiome of *Panax notoginseng* with discrepant yields. Chin Med. 15, 85. 10.1186/s13020-020-00364-4 32793300 PMC7418314

[B34] Liu D. Sun H. Ma H. (2019). Deciphering microbiome related to rusty roots of *Panax ginseng* and evaluation of antagonists against pathogenic ilyonectria. Front Microbiol. 10, 1350. 10.3389/fmicb.2019.01350 31275274 PMC6591430

[B35] Luo L. Guo C. Wang L. Zhang J. Deng L. Luo K. (2019). Negative plant-soil feedback driven by re-assemblage of the rhizosphere microbiome with the growth of *Panax notoginseng*. Front Microbiol. 10, 1597. 10.3389/fmicb.2019.01597 31404300 PMC6676394

[B36] Ma B. Dai Z. Wang H. Dsouza M. Liu X. He Y.. (2017). Distinct biogeographic patterns for archaea, bacteria, and fungi along the vegetation gradient at the continental scale in Eastern China. mSystems 2017, 2. 10.1128/mSystems.00174-16 28191504 PMC5296412

[B37] Ma B. Wang H. Dsouza M. Lou J. He Y. Dai Z. (2016). Geographic patterns of co-occurrence network topological features for soil microbiota at continental scale in eastern China. ISME J. 10, 1891–1901. 10.1038/ismej.2015.261 26771927 PMC5029158

[B38] Mendes L. W. Kuramae E. E. Navarrete A. A. van Veen J. A. Tsai S. M. (2014). Taxonomical and functional microbial community selection in soybean rhizosphere. ISME J. 8, 1577–1587. 10.1038/ismej.2014.17 24553468 PMC4817605

[B39] Mendes L. W. Raaijmakers J. M. de Hollander M. Mendes R. Tsai S. M. (2018). Influence of resistance breeding in common bean on rhizosphere microbiome composition and function. ISME J. 12, 212–224. 10.1038/ismej.2017.158 29028000 PMC5739014

[B40] Meng X. X. Huang L. F. Dong L. L. Li X. W. Wei F. G. Chen Z. J. (2016). Analysis of global ecology of Panax notoginseng in suitability and quality. Yao Xue Xue Bao. 51, 1483–1493. 10.16438/j.0513-4870.2016-0733 29924558

[B41] Mueller R. C. Paula F. S. Mirza B. S. Rodrigues J. L. Nusslein K. Bohannan B. J. (2014). Links between plant and fungal communities across a deforestation chronosequence in the Amazon rainforest. ISME J. 8, 1548–1550. 10.1038/ismej.2013.253 24451208 PMC4069395

[B42] Nilsson R. H. Larsson K.-H. Taylor A. F. S. Bengtsson-Palme J. Jeppesen T. S. Schigel D. (2019). The UNITE database for molecular identification of fungi: handling dark taxa and parallel taxonomic classifications. Nucleic Acids Res. 47, D259–D264. 10.1093/nar/gky1022 30371820 PMC6324048

[B43] Oksanen J. Blanchet G. Friendly M. Kindt R. Legendre P. McGlinn D. vegan: Community Ecology Package. R package version 25-6. (2019).

[B44] Perez-Jaramillo J. E. Mendes R. Raaijmakers J. M. (2016). Impact of plant domestication on rhizosphere microbiome assembly and functions. Plant Mol Biol. 90, 635–644. 10.1007/s11103-015-0337-7 26085172 PMC4819786

[B45] Philippot L. Raaijmakers J. M. Lemanceau P. van der Putten W. H. (2013). Going back to the roots: the microbial ecology of the rhizosphere. Nat Rev Microbiol. 11, 789–799. 10.1038/nrmicro3109 24056930

[B46] Qiao Q. Wang F. Zhang J. Chen Y. Zhang C. Liu G. (2017). The Variation in the Rhizosphere Microbiome of Cotton with Soil Type, Genotype and Developmental Stage. Sci Rep. 7, 3940. 10.1038/s41598-017-04213-7 28638057 PMC5479781

[B47] Quast C. Pruesse E. Yilmaz P. Gerken J. Schweer T. Yarza P. (2013). The SILVA ribosomal RNA gene database project: improved data processing and web-based tools. Nucleic Acids Res. 41, D590–D596. 10.1093/nar/gks1219 23193283 PMC3531112

[B48] Robinson M. D. McCarthy D. J. Smyth G. K. (2010). edgeR: a Bioconductor package for differential expression analysis of digital gene expression data. Bioinformatics. 26, 139–140. 10.1093/bioinformatics/btp616 19910308 PMC2796818

[B49] Rosseel Y. (2012). lavaan: an R package for structural equation modeling. J Stat Softw. 48, 1–36. 10.18637/jss.v048.i0225601849

[B50] Ruiz V. E. Battaglia T. Kurtz Z. D. Bijnens L. Ou A. Engstrand I. (2017). A single early-in-life macrolide course has lasting effects on murine microbial network topology and immunity. Nat. Commun. 8, 518. 10.1038/s41467-017-00531-6 28894149 PMC5593929

[B51] Shi S. Nuccio E. E. Shi Z. J. He Z. Zhou J. Firestone M. K. (2016). The interconnected rhizosphere: High network complexity dominates rhizosphere assemblages. Ecol. Lett. 19, 926–936. 10.1111/ele.12630 27264635

[B52] Smalla K. Wieland G. Buchner A. Zock A. Parzy J. Kaiser S. (2001). Bulk and rhizosphere soil bacterial communities studied by denaturing gradient gel electrophoresis: plant-dependent enrichment and seasonal shifts revealed. Appl. Environ. Microbiol. 67, 4742–4751. 10.1128/AEM.67.10.4742-4751.2001 11571180 PMC93227

[B53] Sugiyama A. Ueda Y. Zushi T. Takase H. Yazaki K. (2014). Changes in the bacterial community of soybean rhizospheres during growth in the field. PLoS ONE. 9, e100709. 10.1371/journal.pone.0100709 24955843 PMC4067361

[B54] Tan Y. Cui Y. Li H. Kuang A. Li X. Wei Y.. (2017). Diversity and composition of rhizospheric soil and root endogenous bacteria in *Panax notoginseng* during continuous cropping practices. J. Basic Microbiol. 57, 337–344. 10.1002/jobm.201600464 28060404

[B55] Team RC. R: A Language and Environment for Statistical Computing. Vienna: R Foundation for Statistical Computing (2019).

[B56] Tkacz A. Cheema J. Chandra G. Grant A. Poole P. S. (2015). Stability and succession of the rhizosphere microbiota depends upon plant type and soil composition. ISME J. 9, 2349–2359. 10.1038/ismej.2015.41 25909975 PMC4611498

[B57] Toju H. Peay K. G. Yamamichi M. Narisawa K. Hiruma K. Naito K. (2018). Core microbiomes for sustainable agroecosystems. Nat. Plants. 4, 247–257. 10.1038/s41477-018-0139-4 29725101

[B58] Uroz S. Buee M. Murat C. Frey-Klett P. Martin F. (2010). Pyrosequencing reveals a contrasted bacterial diversity between oak rhizosphere and surrounding soil. Environ. Microbiol. Rep. 2, 281–288. 10.1111/j.1758-2229.2009.00117.x 23766079

[B59] Wei Z. Yang T. Friman V. P. Xu Y. Shen Q. Jousset A. (2015). Trophic network architecture of root-associated bacterial communities determines pathogen invasion and plant health. Nat. Commun. 6, 8413. 10.1038/ncomms9413 26400552 PMC4598729

[B60] Xiao C. Yang L. Zhang L. Liu C. Han M. (2016). Effects of cultivation ages and modes on microbial diversity in the rhizosphere soil of *Panax ginseng*. J. Ginseng Res. 40, 28–37. 10.1016/j.jgr.2015.04.004 26843819 PMC4703740

[B61] Xu J. Zhang Y. Zhang P. Trivedi P. Riera N. Wang Y. (2018). The structure and function of the global citrus rhizosphere microbiome. Nat. Commun. 9, 4894. 10.1038/s41467-018-07343-2 30459421 PMC6244077

[B62] Yang M. Zhang X. Xu Y. Mei X. Jiang B. Liao J. (2015). Autotoxic ginsenosides in the rhizosphere contribute to the replant failure of *Panax notoginseng*. PLoS ONE. 10, e0118555. 10.1371/journal.pone.0118555 25695831 PMC4335038

[B63] Zhalnina K. Louie K. B. Hao Z. Mansoori N. da Rocha U. N. Shi S.. (2018). Dynamic root exudate chemistry and microbial substrate preferences drive patterns in rhizosphere microbial community assembly. Nat. Microbiol. 3, 470–480. 10.1038/s41564-018-0129-3 29556109

[B64] Zhang G. Wei G. Wei F. Chen Z. He M. Jiao S. (2021a). Dispersal Limitation plays stronger role in the community assembly of fungi relative to bacteria in rhizosphere across the arable area of medicinal plant. Front. Microbiol. 12, 713523. 10.3389/fmicb.2021.713523 34484152 PMC8415459

[B65] Zhang G. Wei G. Wei F. Chen Z. He M. Jiao S. (2021b). Homogeneous selection shapes rare biosphere in rhizosphere of medicinal plant. Ecol. Indic. 129, 107981. 10.1016/j.ecolind.2021.107981

[B66] Zhao Y. P. Lin S. Chu L. Gao J. Azeem S. Lin W. (2016). Insight into structure dynamics of soil microbiota mediated by the richness of replanted *Pseudostellaria heterophylla*. Sci. Rep. 6, 26175. 10.1038/srep26175 27188449 PMC4870612

