# Supplementary material for: Temporal Dynamics of Rhizosphere Communities Across the Life Cycle of Panax notoginseng
Source: Front Microbiol. 2022 Apr 1;13:853077. doi: 10.3389/fmicb.2022.853077 (PMC9010977; doi:10.3389/fmicb.2022.853077)
Supplement: Supplementary file 1 [file Data_Sheet_1.PDF]

Title: Temporal Dynamics of Rhizosphere Communities across the Life Cycle of *Panax notoginseng*

Running title: Temporal Dynamics of Rhizosphere Communities

Guangfei Wei[1,†], Mengzhi Li[1,†], Guozhuang Zhang[1,†], Zhongjian Chen[2], Fugang Wei[3], Shuo Jiao[4], Jun Qian[1], Yong Wang[2], Jianhe Wei[5], Yitao Wang[6], Xiangxiao Meng[1], Marin Fitzgerald[7], Yuqi Yu[3], Linlin Dong[1,*], Shilin Chen [1,*]

[1]*Key Laboratory of Beijing for Identification and Safety Evaluation of Chinese Medicine, Institute of Chinese Materia Medica, China Academy of Chinese Medical Sciences, Beijing 100700, China;*

[2]*Institute of Sanqi Research, Wenshan University, Wenshan 663000, China;*

[3]*Wenshan Miaoxiang Notoginseng Technology, Co., Ltd., Wenshan 663000, China;*

[4]*State Key Laboratory of Crop Stress Biology in Arid Areas, College of Life Sciences, Northwest A & F University, Yangling 712100, China*

[5]*Hainan Provincial Key Laboratory of Resources Conservation and Development of Southern Medicine, Hainan Branch of the Institute of Medicinal Plant Development, Chinese Academy of Medical Sciences and Peking Union Medical College, Haikou 570311, China*

[6]*State Key Laboratory of Quality Research in Chinese Medicine, Institute of Chinese Medical Sciences, University of Macau, Taipa, Macao 999077, China*

[7]*Department of Life Sciences, University of Westminster, London 1W1W6UW, England*

E-mail addresses:

Guangfei Wei: gfwei@icmm.ac.cn

Mengzhi Li: mengzhili0124@163.com

Guozhuang Zhang: zgz123zgz@163.com

Zhongjian Chen: 18687656337@126.com

Fugang Wei: weifugang@live.com

Shuo Jiao: shuojiao@nwsuaf.edu.cn

Jun Qian: qianjun@biozeron.com

Yong Wang: ws-wangyong37@163.com

Jianhe Wei: wjianh@263.net

Yitao Wang: ytwang@um.edu.mo

Xiangxiao Meng: xxmeng@icmm.ac.cn

Marin Fitzgerald: M.fitzgerald@my.westminster.ac.uk

Yuqi Yu: yu-yu-qi@163.com

Author contributions

[†]Contributed equally to this work.

[*]**Corresponding authors:** Linlin Dong and Shilin Chen

Tel.: +86-10-5720-3877 (Linlin Dong); Fax: +86-10-6289-9776 (Shilin Chen)

Email: lldong@icmm.ac.cn (Linlin Dong); slchen@icmm.ac.cn (Shilin Chen)

Supporting Information

A. Supporting Tables

Table S1 The location of sites for temporal sampling and corresponding edaphic and climatic factors

Table S2 Statistics in redundancy analysis (RDA) of microbial communities collected from different developmental stages.

B. Supporting Figures

Figure S1 Shifts in the compositions of bacterial and fungal communities as *P. notoginseng* growth

Figure S2 Differential abundance test of bacterial and fungal genus between each stages and the bulk soils

Figure S3 The bacterial and fungal subnetworks of each stage. The colors of nodes represent network modules

Figure S4 The principal coordinate analysis of bacterial and fungal communities (a) as well as the principal component analysis of edaphic factors (b).

Table S1 The location of sites for temporal sampling and corresponding edaphic and climatic factors

Sample	Stage	MMT	AK (mg/kg)	OM (g/kg)	AP (mg/kg)	TN (g/kg)	PH	site	Latitude	Longitude	Sample	Stage	MMT	AK (mg/kg)	OM (g/kg)	AP (mg/kg)	TN (g/kg)	PH	site	Latitude	Longitude
PBAFL1	FL	13.6	205.98	36.13	13.29	0.5	5.82	PBA	23.3167	104.2025	QBFL1	FL	11.5	489.8	57.32	33	0.64	4.77	QB	23.8297	104.1036
PBAFL2	FL	13.6	196.19	35.99	14.68	0.36	5.66	PBA	23.3167	104.2025	QBFL2	FL	11.5	401.72	57.44	37.65	0.78	4.81	QB	23.8297	104.1036
PBAFL3	FL	13.6	196.19	33.48	13.22	0.36	5.59	PBA	23.3167	104.2025	QBFL3	FL	11.5	421.29	54.49	36.76	0.64	4.66	QB	23.8297	104.1036
PBA2YV1	2YV	22.2	215.77	29.86	66.13	0.36	6.26	PBA	23.3167	104.2025	QB2YV1	2YV	20.9	431.08	47.22	67.14	0.36	5.85	QB	23.8297	104.1036
PBA2YV2	2YV	22.2	225.55	24.43	62.3	0.36	6.98	PBA	23.3167	104.2025	QB2YV2	2YV	20.9	401.72	37.55	68.08	0.5	5.25	QB	23.8297	104.1036
PBA2YV3	2YV	22.2	294.06	34.91	63.44	0.5	6.35	PBA	23.3167	104.2025	QB2YV3	2YV	20.9	382.15	45.51	72.53	0.36	5.98	QB	23.8297	104.1036
PBA2YF1	2YF	23	342.99	31.8	51.89	0.5	6.12	PBA	23.3167	104.2025	QB2YF1	2YF	21.9	538.74	50.06	80.64	0.78	5.51	QB	23.8297	104.1036
PBA2YF2	2YF	23	313.64	28.33	64.28	0.5	6.86	PBA	23.3167	104.2025	QB2YF2	2YF	21.9	480.02	57.23	79.7	0.36	5.76	QB	23.8297	104.1036
PBA2YF3	2YF	23	401.72	37.55	66.13	0.52	6.43	PBA	23.3167	104.2025	QB2YF3	2YF	21.9	431.08	67.55	69.37	1.34	5.08	QB	23.8297	104.1036
PBA2YR1	2YR	18.8	411.51	31.79	71.82	0.5	6.04	PBA	23.3167	104.2025	QB2YR1	2YR	16.8	401.72	51.48	81.9	0.5	6.01	QB	23.8297	104.1036
PBA2YR2	2YR	18.8	342.99	24.55	55.6	0.64	6.19	PBA	23.3167	104.2025	QB2YR2	2YR	16.8	401.72	71.79	91.55	0.36	5.94	QB	23.8297	104.1036
PBA2YR3	2YR	18.8	391.93	35.92	71.35	0.22	6.23	PBA	23.3167	104.2025	QB2YR3	2YR	16.8	470.23	71.94	90.11	0.5	5.93	QB	23.8297	104.1036
PBA3YV1	3YV	22.2	284.28	30.43	35.45	0.36	6.06	PBA	23.3167	104.2025	QB3YV1	3YV	20.9	323.42	43.61	74.34	0.5	5.69	QB	23.8297	104.1036
PBA3YV2	3YV	22.2	303.85	31.79	40.37	0.22	5.88	PBA	23.3167	104.2025	QB3YV2	3YV	20.9	450.66	50.57	92.31	0.36	5.73	QB	23.8297	104.1036
PBA3YV3	3YV	22.2	323.42	36.87	53.79	0.36	5.81	PBA	23.3167	104.2025	QB3YV3	3YV	20.9	333.21	51.58	76.81	0.36	5.49	QB	23.8297	104.1036
PBA3YF1	3YF	23	323.42	46.33	55.35	0.36	5.51	PBA	23.3167	104.2025	QB3YF1	3YF	21.9	303.85	62.31	76.76	0.36	5.13	QB	23.8297	104.1036
PBA3YF2	3YF	23	205.98	35.43	55.33	0.36	6.04	PBA	23.3167	104.2025	QB3YF2	3YF	21.9	362.57	79.76	75.3	0.5	4.79	QB	23.8297	104.1036
PBA3YF3	3YF	23	235.34	47.15	64.1	0.5	6.36	PBA	23.3167	104.2025	QB3YF3	3YF	21.9	313.64	78.84	73.79	0.64	4.81	QB	23.8297	104.1036
PBA3YR1	3YR	18.8	215.77	28.62	20.02	0.64	6.77	PBA	23.3167	104.2025	QB3YR1	3YR	16.8	284.28	64.63	69	0.36	5.39	QB	23.8297	104.1036
PBA3YR2	3YR	18.8	147.26	25.74	17.25	0.78	6.23	PBA	23.3167	104.2025	QB3YR2	3YR	16.8	254.92	53.39	75.08	0.36	5.44	QB	23.8297	104.1036
PBA3YR3	3YR	18.8	205.98	34.14	17.25	0.64	6.14	PBA	23.3167	104.2025	QB3YR3	3YR	16.8	323.42	60.17	68.01	0.36	5.21	QB	23.8297	104.1036
PBBFL1	FL	13.6	274.49	40.29	14.31	0.5	5.56	PBB	23.2097	104.1342	PBCFL1	FL	13.6	313.64	53.01	27.41	0.36	4.86	PBC	23.2415	104.0842
PBBFL2	FL	13.6	245.13	40.29	14.73	0.36	5.51	PBB	23.2097	104.1342	PBCFL2	FL	13.6	313.64	53.73	24.57	0.5	4.71	PBC	23.2415	104.0842

PBBFL3	FL	13.6	254.92	44.92	14.31	0.36	5.43	PBB	23.2097	104.1342	PBCFL3	FL	13.6	303.85	53.49	24.3	0.64	4.59	PBC	23.2415	104.0842
PBB2YV1	2YV	22.2	284.28	35.91	26.37	0.64	6.05	PBB	23.2097	104.1342	PBC2YV1	2YV	22.2	235.34	41.87	48.8	1.34	5.42	PBC	23.2415	104.0842
PBB2YV2	2YV	22.2	264.71	40.03	29.12	0.22	5.92	PBB	23.2097	104.1342	PBC2YV2	2YV	22.2	362.57	59.05	66.75	2.18	5.76	PBC	23.2415	104.0842
PBB2YV3	2YV	22.2	225.55	36.5	19.72	0.36	5.71	PBB	23.2097	104.1342	PBC2YV3	2YV	22.2	235.34	57.23	41.88	0.78	5.16	PBC	23.2415	104.0842
PBB2YF1	2YF	23	440.87	40.96	43.98	0.22	6.22	PBB	23.2097	104.1342	PBC2YF1	2YF	23	411.51	60.99	75.38	0.5	6.02	PBC	23.2415	104.0842
PBB2YF2	2YF	23	431.08	43.97	51.62	0.36	6.01	PBB	23.2097	104.1342	PBC2YF2	2YF	23	342.99	60.87	62.67	0.36	5.57	PBC	23.2415	104.0842
PBB2YF3	2YF	23	401.72	43.97	49.54	0.36	6.26	PBB	23.2097	104.1342	PBC2YF3	2YF	23	382.15	44.88	73.5	0.36	6.14	PBC	23.2415	104.0842
PBB2YR1	2YR	18.8	391.93	36.37	64.99	0.64	5.92	PBB	23.2097	104.1342	PBC2YR1	2YR	18.8	313.65	53.14	76.12	0.36	6.06	PBC	23.2415	104.0842
PBB2YR2	2YR	18.8	391.93	41.24	54.04	0.36	5.71	PBB	23.2097	104.1342	PBC2YR2	2YR	18.8	313.64	59.03	72.98	0.36	6.53	PBC	23.2415	104.0842
PBB2YR3	2YR	18.8	372.36	44.48	53.97	0.5	5.64	PBB	23.2097	104.1342	PBC2YR3	2YR	18.8	254.92	57.09	76.61	0.5	5.78	PBC	23.2415	104.0842
PBB3YV1	3YV	22.2	342.99	35.05	42	0.22	5.85	PBB	23.2097	104.1342	PBC3YV1	3YV	22.2	245.13	48.31	69.52	0.64	5.43	PBC	23.2415	104.0842
PBB3YV2	3YV	22.2	313.64	39.66	45.63	0.22	5.87	PBB	23.2097	104.1342	PBC3YV2	3YV	22.2	176.62	50.27	49.99	0.5	5.41	PBC	23.2415	104.0842
PBB3YV3	3YV	22.2	342.99	38.38	44.94	0.36	5.69	PBB	23.2097	104.1342	PBC3YV3	3YV	22.2	235.34	36.72	47.09	0.64	5.76	PBC	23.2415	104.0842
PBB3YF1	3YF	23	294.06	55.98	67.24	0.64	6.19	PBB	23.2097	104.1342	PBC3YF1	3YF	23	245.13	76.91	89.62	0.22	5.52	PBC	23.2415	104.0842
PBB3YF2	3YF	23	235.34	56.67	41.03	0.36	5.92	PBB	23.2097	104.1342	PBC3YF2	3YF	23	176.62	72.82	62.2	0.5	5.15	PBC	23.2415	104.0842
PBB3YF3	3YF	23	225.55	54.05	37.57	0.22	5.81	PBB	23.2097	104.1342	PBC3YF3	3YF	23	196.19	64.37	78.62	0.22	5.66	PBC	23.2415	104.0842
PBB3YR1	3YR	18.8	196.19	43.24	23.28	0.22	5.74	PBB	23.2097	104.1342	PBC3YR1	3YR	18.8	147.29	61.89	32.63	0.36	4.65	PBC	23.2415	104.0842
PBB3YR2	3YR	18.8	205.98	40.35	23.09	0.36	5.65	PBB	23.2097	104.1342	PBC3YR2	3YR	18.8	147.29	60.27	38.54	0.64	4.61	PBC	23.2415	104.0842
PBB3YR3	3YR	18.8	215.77	41.46	26.3	0.36	5.61	PBB	23.2097	104.1342	PBC3YR3	3YR	18.8	186.41	59.72	50.8	0.5	4.63	PBC	23.2415	104.0842
YSFL1	FL	11.4	313.64	48.47	24.91	0.5	5.52	YS	23.5823	104.3303											
YSFL2	FL	11.4	323.42	44.75	24.84	0.64	5.62	YS	23.5823	104.3303											
YSFL3	FL	11.4	333.21	48.47	24.94	0.36	5.63	YS	23.5823	104.3303											
YS2YV1	2YV	20	352.76	47.24	30.9	0.36	6.38	YS	23.5823	104.3303											
YS2YV2	2YV	20	294.06	48.71	26.3	1.2	6.34	YS	23.5823	104.3303											
YS2YV3	2YV	20	382.15	52.93	32.18	0.64	6.23	YS	23.5823	104.3303											
YS2YF1	2YF	21	372.36	53.39	34.85	1.2	6.38	YS	23.5823	104.3303											

YS2YF2	2YF	21	421.29	55.31	49.44	1.76	6.51	YS	23.5823	104.3303
YS2YF3	2YF	21	460.44	53.17	45.73	1.48	6.03	YS	23.5823	104.3303
YS2YR1	2YR	16.6	401.72	46.73	41.53	0.22	6.23	YS	23.5823	104.3303
YS2YR2	2YR	16.6	362.57	49.73	32.23	0.5	6.11	YS	23.5823	104.3303
YS2YR3	2YR	16.6	372.36	54.48	40.94	0.64	6.15	YS	23.5823	104.3303
YS3YV1	3YV	20	431.08	47.59	47.31	0.36	5.96	YS	23.5823	104.3303
YS3YV2	3YV	20	411.51	51.89	52.24	0.36	6.14	YS	23.5823	104.3303
YS3YV3	3YV	20	431.08	50.22	54.36	0.22	6.01	YS	23.5823	104.3303
YS3YF1	3YF	21	352.79	69.22	48.5	0.5	6.15	YS	23.5823	104.3303
YS3YF2	3YF	21	323.42	69.95	51.54	0.5	5.89	YS	23.5823	104.3303
YS3YF3	3YF	21	284.28	70.16	47.46	0.5	5.68	YS	23.5823	104.3303
YS3YR1	3YR	16.6	294.06	52.17	37.1	0.22	6.07	YS	23.5823	104.3303
YS3YR2	3YR	16.6	352.79	54.62	45.14	0.5	6.03	YS	23.5823	104.3303
YS3YR3	3YR	16.6	362.57	61.41	56.17	0.5	6.09	YS	23.5823	104.3303

Table S2 Statistics in redundancy analysis (RDA) of microbial communities collected from different developmental stages. MMT, month mean temperature; AK, available potassium; OM, organic matter; AP, available phosphate; TN, total nitrogen.

	Influence factors	Variance (%)	*P* values	Significance
Bacteria	Developmental stage	6.76	0.001	***
	AK	1.82	0.001	***
	OM	1.61	0.001	***
	AP	1.89	0.001	***
	TN	0.35	0.097	-
	pH	2.19	0.001	***
	MMT	2.45	0.001	***
Fungi	Developmental stage	5.26	0.001	***
	AK	2.71	0.001	***
	OM	1.66	0.001	***
	AP	2.68	0.001	***
	TN	0.42	0.154	-
	pH	2.24	0.001	***
	MMT	3.06	0.001	***

"*", "**" and "***" represent significance level of 0.05, 0.01 and 0.001, respectively.

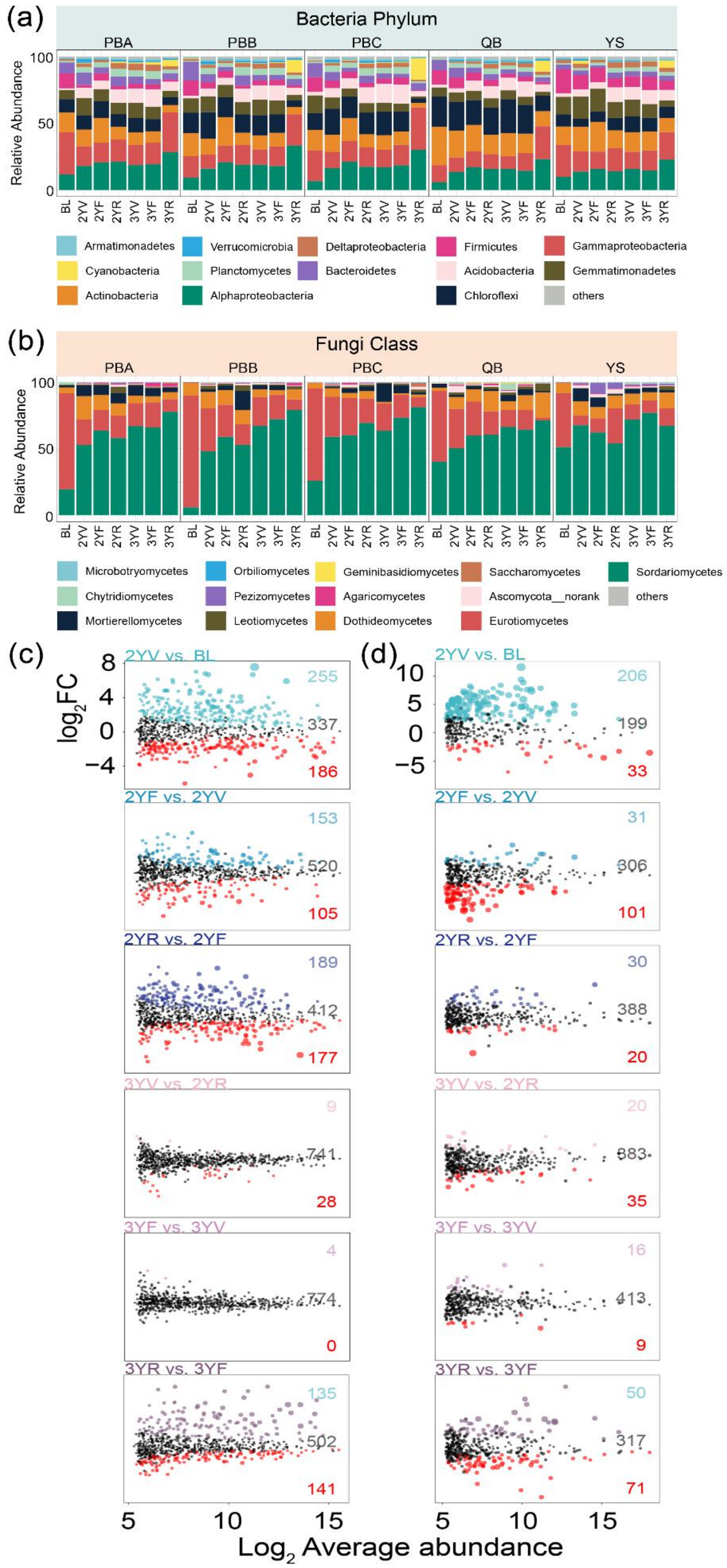

Figure S1 Shifts in the compositions of bacterial and fungal communities as *P. notoginseng* growth. Bar plots of top 13 phyla relative abundance of bacteria (a) and top 13 class of fungi (b).

Enrichment and depletion of bacterial (c) and fungal (d) genus for each growth stages compared to last sampling time point. Each point in the scatterplot represents an individual genus, the size of point is mapped to the minus logarithmic transformation of FDR adjusted *p*-values while the positions along *x* and *y* axis represent average relative abundance and abundance fold change respectively. BL, 2YV, 2YF, and 2YR represent bulk soils and the 2-year vegetative, flowering, and root growth stages, respectively. 3YV, 3YF, and 3YR denote the 3-year vegetative, flowering, and root growth stages, respectively.

At the phylum level of bacteria, the relative abundance of Alphaproteobacteria, Acidobacteria, and Planctomycetes increased consistently from samples of BL stage to 3YF stage, while the abundance of Firmicutes and Bacteroidetes decreased (Figure S1a). Compared to soils of 3YF stage, the relative abundance of Alphaproteobacteria, Gammaproteobacteria, and Cyanobacteria showed dramatical increase while the abundance of Actinobacteria, Gemmatimonadetes, Firmicutes, and Chloroflexi displayed marked reduction in the soils of 3YR stage. At the class level of fungi, the relative abundance of Sordariomycetes showed increasing trend, while Eurotiomycetes appeared a decreasing trend after *P. notoginseng* transplanted (Figure S1b). The significant enrichment and depletion of bacteria were active in the *P. notoginseng* rhizosphere at 2YV, 2YF, 2YR, and 3YR stages, with 255, 153, 189, and 135 genera increased, and 186, 105, 177, and 141 decreased, respectively (Figure S1c). Fungal genera were mainly enriched and depleted at the 3YR stage, and kept stable from 2YF stage to 3YF stage (Figure S1d).

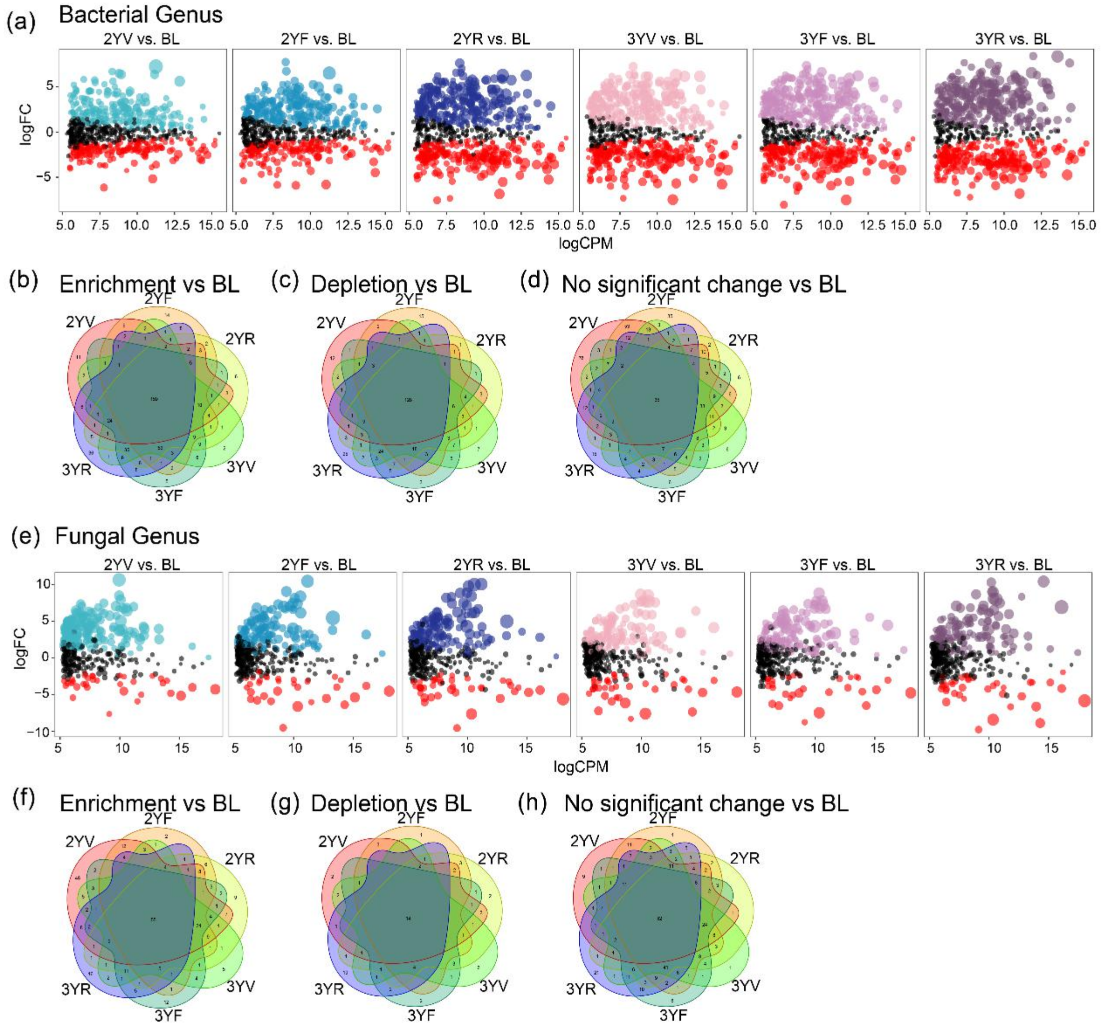

Fig. S2 Differential abundance test of bacterial and fungal genus between each stage and the bulk soils. Differential abundance test of bacterial genus (a) and fungal genus (e). Red points represent bacterial genus which are significantly enriched in the bulk soils. Black points represent bacterial and fungal genus which have no significant difference in abundance. Venn plots show the number of shared significantly enriched (b, f), significantly depleted (c, g) and no significantly changed (d, h). BL, 2YV, 2YF, and 2YR represent bulk soils and the 2-year vegetative, flowering, and root growth stages, respectively. 3YV, 3YF, and 3YR denote the 3-year vegetative, flowering, and root growth stages, respectively.

Differential abundance test of each developmental stage compared with BL was carried out to analyze the enriched and depleted communities during the growth of *P. notoginseng* (Figure S2). More bacterial genera were enriched and depleted as plant growth, with 159 and 128 genera showing consistent significant increase and decrease from 2YV stage to 3YR stage, respectively.

Only 35 in 778 bacterial genera kept stable abundance during different stages (Figure S2a-2d). In contrast, less fungal genera were enriched as plant growth, and there were 82 in 438 fungal genera maintaining stable abundance (Figure S2e-2h). These results indicated that the composition of rhizosphere microbiomes was shaped by developmental stages.

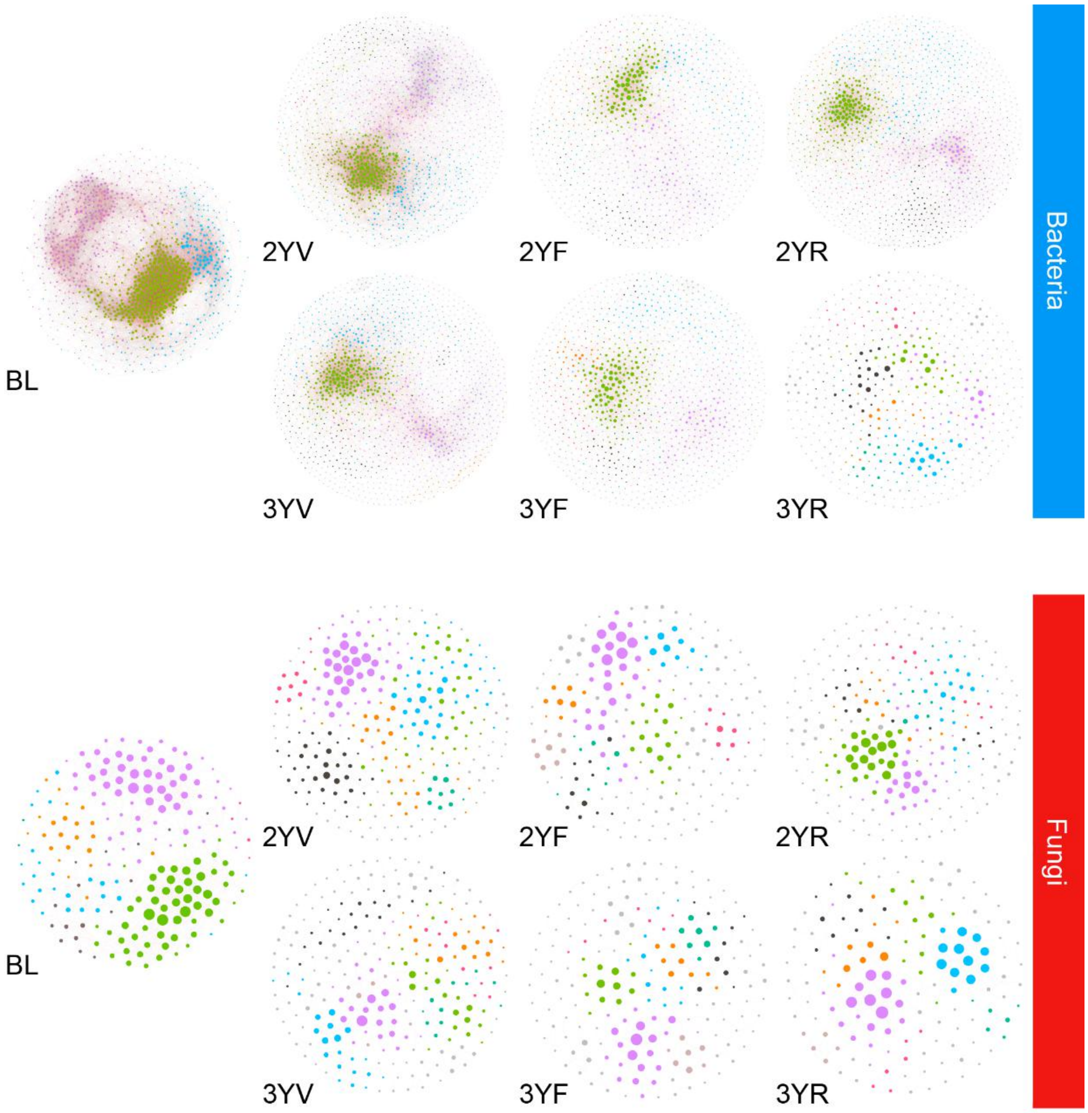

Fig. S3 The bacterial and fungal subnetworks of each stage. The colors of nodes represent network modules.

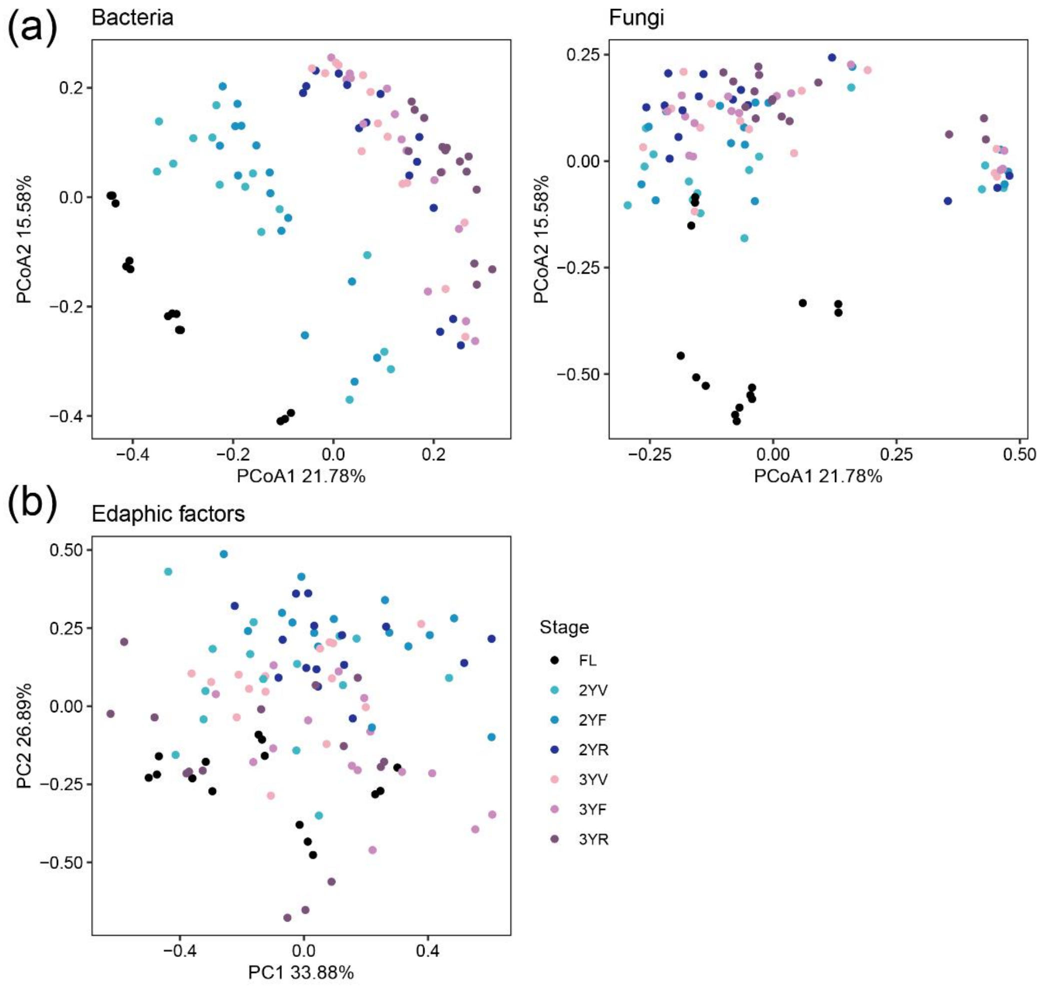

Figure S4 The principal coordinate analysis of bacterial and fungal communities (a) as well as the principal component analysis of edaphic factors (b).
